# The noisy encoding of disparity model predicts perception of the McGurk effect in native Japanese speakers

**DOI:** 10.3389/fnins.2024.1421713

**Published:** 2024-06-26

**Authors:** John F. Magnotti, Anastasia Lado, Michael S. Beauchamp

**Affiliations:** Department of Neurosurgery, Perelman School of Medicine, University of Pennsylvania, Philadelphia, PA, United States

**Keywords:** audiovisual, speech, multisensory, cross-cultural, illusion

## Abstract

In the McGurk effect, visual speech from the face of the talker alters the perception of auditory speech. The diversity of human languages has prompted many intercultural studies of the effect in both Western and non-Western cultures, including native Japanese speakers. Studies of large samples of native English speakers have shown that the McGurk effect is characterized by high variability in the susceptibility of different individuals to the illusion and in the strength of different experimental stimuli to induce the illusion. The noisy encoding of disparity (NED) model of the McGurk effect uses principles from Bayesian causal inference to account for this variability, separately estimating the susceptibility and sensory noise for each individual and the strength of each stimulus. To determine whether variation in McGurk perception is similar between Western and non-Western cultures, we applied the NED model to data collected from 80 native Japanese-speaking participants. Fifteen different McGurk stimuli that varied in syllable content (unvoiced auditory “pa” + visual “ka” or voiced auditory “ba” + visual “ga”) were presented interleaved with audiovisual congruent stimuli. The McGurk effect was highly variable across stimuli and participants, with the percentage of illusory fusion responses ranging from 3 to 78% across stimuli and from 0 to 91% across participants. Despite this variability, the NED model accurately predicted perception, predicting fusion rates for individual stimuli with 2.1% error and for individual participants with 2.4% error. Stimuli containing the unvoiced pa/ka pairing evoked more fusion responses than the voiced ba/ga pairing. Model estimates of sensory noise were correlated with participant age, with greater sensory noise in older participants. The NED model of the McGurk effect offers a principled way to account for individual and stimulus differences when examining the McGurk effect in different cultures.

## Introduction

In the McGurk effect, pairing an auditory syllable with an incongruent visual syllable produces the percept of a third syllable different than either component syllable (McGurk and MacDonald, 1976). The illusion demonstrates the powerful influence of visual information on auditory speech perception and has become a popular instrument for examining audiovisual integration (Beauchamp, 2018).

Several models have been developed to account for various properties of the McGurk effect. The pioneering fuzzy logic model of Massaro (1998) was developed to explain why some pairings of incongruent audiovisual combinations produce an illusory fusion percept, but for most, participants report the auditory component of the stimulus. More recent models improve on the fuzzy logic model (Schwartz, 2010) often by incorporating principles of Bayesian inference (Andersen and Winther, 2020; Lindborg and Andersen, 2021) and causal inference (Magnotti et al., 2013, 2018; Magnotti and Beauchamp, 2017), although models featuring dynamic predictive mechanisms (Olasagasti et al., 2015) and parallel linear dynamic processes (Altieri and Yang, 2016) have also been proposed.

One challenge to modeling studies of the McGurk effect is the relatively recent realization that there is enormous variability in the McGurk effect across experimental stimuli and participants (Schwartz, 2010; Jiang and Bernstein, 2011; Stevenson et al., 2012; Basu Mallick et al., 2015). The original description of the McGurk effect used stimuli recorded from a single talker and reported that the illusion was experienced by nearly all participants. In contrast, Basu Mallick et al. tested 12 different McGurk stimuli used in published studies and found that the efficacy of the different stimuli ranged from 17 to 58%. Across participants, some never perceived the illusion (0%) while others perceived the illusion on every presentation of every stimulus (100%) (Basu Mallick et al., 2015).

The noisy encoding of disparity (NED) model was developed in response to this observation of high variability. Rather than attempt to model the perceptual processes that produce the McGurk effect (as in the models described above), the NED model uses Bayesian, probabilistic inference to predict variation in the McGurk effect across stimuli and participants. Stimulus differences are modeled using a single parameter for each stimulus (the audiovisual disparity inherent in the stimulus), while participant differences are modeled with two parameters for each participant (an audiovisual disparity threshold and a sensory noise measure).

Using three parameters, the NED model was able to accurately predict perception in a sample of 165 native English speakers (Magnotti and Beauchamp, 2015). NED also accurately modeled perceptual differences between 8 adult cochlear implant users and 24 normal-hearing subjects who were native German speakers (Stropahl et al., 2017). The NED model validated the experimental prediction of stronger audiovisual integration in cochlear implant users while controlling for differences between stimuli.

Both previous studies that applied the NED model used participants from Western cultures (native speakers of English and German, respectively). However, perception of the McGurk effect has been reported to be markedly reduced in native Japanese speakers (Sekiyama and Tohkura, 1991, 1993). This raised the question of whether the underlying assumptions of the NED model generalize from speakers of Western languages to native speakers of Japanese. To answer this question, we measured the percepts of 80 native Japanese-speaking participants presented with 15 different McGurk stimuli and assessed the fit of the NED model.

## Methods

All experiments were approved by the Institutional Review Board of the University of Pennsylvania, Philadelphia, PA. Data were collected from 101 Japanese-speaking participants recruited from the communities of the Okinawan Institute of Science and Technology, Kyoto University, and the University of Tokyo. All data and analysis code are available in Supplementary material. Participants received an Amazon gift card for ¥2000 upon completion of the experiment.

A total of 240 audiovisual stimuli were presented to each participant in pseudorandom order. The primary stimulus set consisted of 15 McGurk videos each containing the incongruent pairing of auditory *ba* with visual *ga* (AbaVga) or auditory *pa* with visual *ka* (ApaVka; Table 1). Each McGurk video was presented 10 times to each participant. As a control, each participant was also presented with 90 congruent audiovisual stimuli (30 different stimuli presented three times each). Each of the 30 congruent stimuli was recorded from a different talker (9 male, 21 female, no overlap with the McGurk talkers). The syllable composition of the congruent stimuli was 5 AbaVba, 5AgaVga, 5 AdaVda, 5 ApaVpa, 5 AkaVka, and 5 AtaVta.

**Table 1:** Participants were presented with 15 different McGurk stimuli.

Rank	Aud	Vis	Fusion percept	Auditory percept	Voiced/unvoiced	Talker native language is Japanese?	Source
1	baba	gaga	dada	baba	V	no	http://youtu.be/5Lq26mgFpOc
2	baba	gaga	dada	baba	V	no	http://youtu.be/tUf0672xAOU
3	ba	ga	da	ba	V	yes	Kaoru Sekiyama
4	ba	ga	da	ba	V	no	http://youtu.be/WK3T7LWIkP8
5	ba	ga	da	ba	V	no	(Quinto et al., 2010)
6	ba	ga	da	ba	V	no	Kaoru Sekiyama
7	baba	gaga	dada	baba	V	no	http://youtu.be/aFPtc8BVdJk
8	ba	ga	da	ba	V	no	http://youtu.be/rIWrnJH2jAY
9	ba	ga	da	ba	V	yes	Kaoru Sekiyama
10	ba	ga	da	ba	V	no	Kaoru Sekiyama
11	ba	ga	da	ba	V	no	(Dodd et al., 2008)
12	ba	ga	da	ba	V	no	http://youtu.be/jtsfidRq2tw
13	papa	kaka	tata	papa	U	no	Arnt Maaso
14	pa	ka	ta	pa	U	no	http://youtu.be/An5vvn-gcwA
15	pa	ka	ta	pa	U	no	http://youtu.be/51lVOJ8jfxA

Stimuli were ranked based on their strength in evoking the McGurk fusion percept from weakest (1) to strongest (15).

### Experimental procedures and data analysis

**Figure 1:**
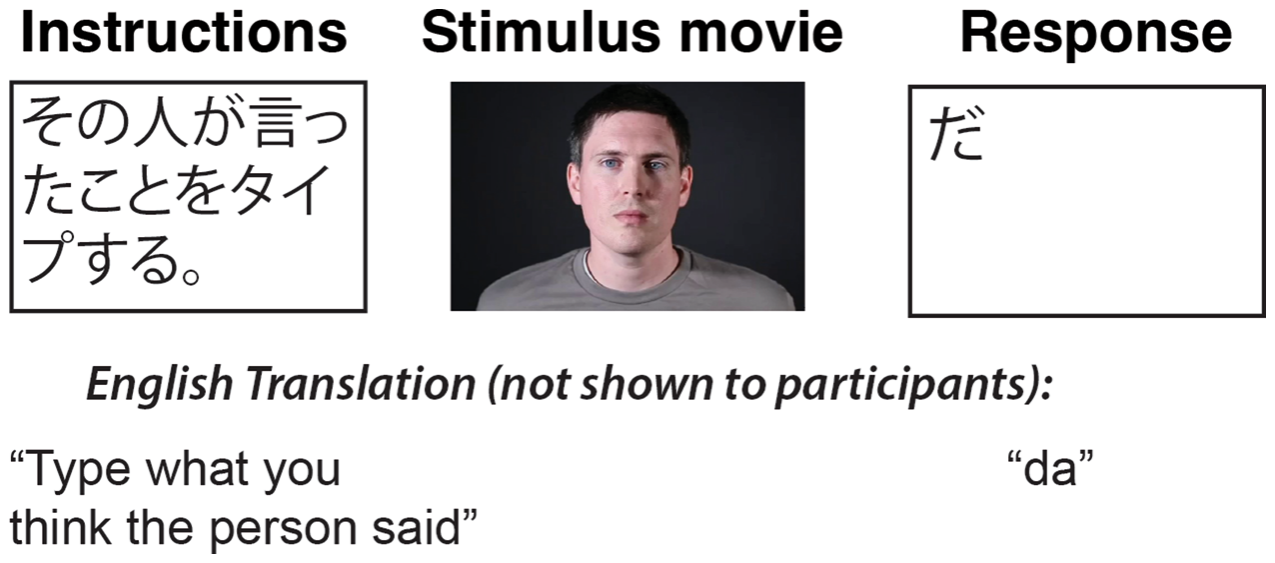
Participants were instructed to report their percept of audiovisual movies containing McGurk and congruent syllables (complete instructions shown in Table 2). On each trial, a stimulus movie was played, and participants entered their response using a free-text response box.

The study was conducted online using the SoSci Survey research platform (Information about SoSci Survey, n.d.). At the beginning of the experiment, all participants received a short description of the study in Japanese (Table 2). Figure 1 shows the structure of each trial. An instruction screen was presented and remained on screen while the stimulus video played. At the conclusion of the video, a response text box appeared that accepted a variety of characteristics, including the Latin alphabet, hiragana, and katakana (sets of symbols used to represent Japanese syllable-like morae).

**Table 2:** Task instructions were presented in Japanese to participants before the beginning of the experiment (first row) and during each trial (second row).

Japanese language instructions	English translation
様々な音節を発音する人々のビデオが流れます。 この人たちが何を言っているのか、回答してください。 必ず静かな環境で、またはノイズキャンセリングヘッドホンを着用してビデオを 見てください。 携帯電話ではなく、必ずノートパソコンまたはPCでビデオを見てください。	A video will show people pronouncing different syllables. Please, type what these people are saying. Please, watch the videos in a quiet environment or wear noise-cancelling headphones. Please, watch the videos on a laptop or PC, not a mobile phone.
ビデオのフレーム全体(上のボックス)が見えるように、ブラウザのウィンドウを調整してください。 上の再生ボタンを押してから、その人が何と言ったか、あなたの考えを下のボックスに入力してください。 間違った答えはありませんので、何度も聴いたり見たりする必要はありません。	Adjust your browser window so that you can see the entire frame of the video. Press the play button above and then type what you think the person said in the box below. There are no wrong answers, so please, play the video only once.

The English translation was not shown to the participants.

Fusion responses to the McGurk videos were defined as “da,” “ta,” or “tha.” Responses were translated into English and assigned to one of four mutually exclusive categories: auditory responses, visual responses, fusion responses, and other responses (e.g., “ha,” “va”). For double syllable stimuli, each syllable received a half-point rating. For example, the response “dada” was given a score of 1.0 as a complete fusion response, while “bada” was rated as 0.5 for auditory and 0.5 for fusion.

Participants with less than 90% accuracy for congruent syllable recognition were excluded from the analysis, ensuring that accuracy for congruent syllables in the remaining participants was high (mean = 96%, standard error of the mean across participants, SEM = 0.3%, range = 90 to 100%). Congruent stimuli almost never evoked fusion responses (2 out of 7,200 trials, 0.03%).

Excluding participants with low congruent accuracy left 80 participants whose data are reported in the manuscript. For these participants, 36 self-reported as male and 44 as female with a mean age of 29 years (range from 18 to 63 years). All participants completed high school and 80% had also completed at least a professional or bachelor’s degree. All participants reported their native language as Japanese. Participants were asked to rate their level of English proficiency on a 0 to 6 scale (0 meaning no proficiency at all); the mean proficiency was 3.0. All participants reported normal hearing and normal or corrected-to-normal vision.

### Noisy encoding of disparity model

The noisy encoding of disparity (NED) model was fit as described in the study by Magnotti and Beauchamp (2015). All analysis code is available in Supplementary material. The model calculates the long-run probability of a fusion percept for each participant and stimulus as follows:


$$P\left(\mathrm{Fusion}\right)=P_{\mathrm{Norm}}\left(X<T\;|\;D;\sigma \right)$$

where *X* is the disparity encoded by the participant on an individual trial. The model parameters are *D*, the stimulus disparity; *T*, the participant’s disparity threshold; and *σ*, the participant’s sensory noise level (the standard deviation of the sensory encoding distribution). The best-fitting parameters were determined by minimizing the error between the model’s predictions and the fusion rate measured for each participant for each stimulus. Error was calculated as the mean absolute error (MAE) for individual subjects across stimuli, individual stimuli across subjects, and for each subject-by-stimulus combination.

In more detail, *D_i_* (where *i* is the stimulus index) captures the likelihood that the auditory and visual component stimulus *i* produce the McGurk effect, which is averaged across all presentations of stimulus *i* to all participants. *T_j_* (where *j* is the participant index) describes each participant’s prior probability for fusing the auditory and visual components of any stimulus. If the disparity of a stimulus (as estimated by the participant in a single trial) is less than *T_j_*, the auditory and visual speech cues are fused, and the participant reports a fusion percept. If the estimated disparity is greater than *T_j_*, the participant reports the auditory components of the stimulus. The estimated disparity is not always equal to *D* because auditory and visual speech features are measured with noise (Ma et al., 2009; Bejjanki et al., 2011), resulting in variability in the measured stimulus disparity across multiple presentations of the same stimulus. Across many trials, the distribution of measured strengths will be Gaussian in shape, centered at the true stimulus disparity, *D_i_* with standard deviation equal to the participant’s sensory noise, *σ_j_*. The amount of sensory noise is assumed to be constant across stimuli for each participant.

The probability of a fusion percept for subject *j* on stimulus *i* is as follows:


$$p\left(x<T_{j}|D_{i}\right)=\overset{T_{j}}{\underset{-\infty }{\int }}N\left(x;\;D_{i},\;\sigma_{j}\right)\;dx$$

where *N* is the normal (Gaussian) distribution with mean *D_i_* and standard deviation *σ_j_*. The invariance of the disparity threshold and sensory noise across stimuli allows the model to predict a participant’s fusion proportion for any stimulus with known strength, even if the participant has not observed the stimulus. Because the stimulus disparities are fixed across participants, they cannot fit participant variability; stimulus disparities and participant disparity thresholds are independent.

### Generalization testing

To assess the generalizability of the model results, a hold-out procedure was implemented. For each participant, the NED model was fit without that participant’s data to obtain stimulus disparity values. Next, holding out a single stimulus, the best-fitting threshold and sensory noise parameters were determined for the held-out participant (without using data from the held-out stimulus). Using the fitted subject-level parameters and the stimulus disparity values obtained from other participants, the participant’s fusion perception was predicted for the held-out stimulus. This procedure was repeated for each stimulus, resulting in a predicted fusion response for each stimulus that was unbiased by the participant’s data for that stimulus. The result of this procedure is a predicted fusion proportion for each participant for each stimulus.

### Predictors of participant and stimulus variability

Multiple linear regression models were constructed to examine the relationship between participant-level model parameters (one model for disparity threshold, one for sensory noise) and participant demographic variables (age, gender, English proficiency, and highest education level). The models were obtained using stepwise regression to automatically select the best parameters using the Bayesian Information Criterion (BIC) cost function. Because of the small sample size, no interactions were allowed during the stepwise model building procedure. The initial model was the model including all variables, and the minimal model was the intercept-only model.

To understand stimulus-level variation, the same procedure was applied to the stimulus parameter of disparity, with stimulus variables of syllabic content (voiced AbaVga vs. voiceless ApaVka), talker gender (male vs. female), and talker native language (Japanese vs. non-Japanese).

### Stimulus details

The McGurk stimuli used in the original description of the effect are lost to history (MacDonald, 2018). To sample currently available McGurk stimuli, 15 different stimuli (s1–s15) were collected from popular online demonstrations and previously published studies (Table 1). Previous in-person and online studies presented many of the same stimuli (Basu Mallick et al., 2015; Magnotti et al., 2015, 2024).

The stimuli were made at different times by different groups using different methods. For the details of stimulus creation for s2, see Green and Kuhl (1989, 1991); for s3, s6, s9, s10, see Sekiyama (1994, 1997); Sekiyama and Tohkura (1991, 1993); for s4 and s14, see Nath et al. (2011); Nath and Beauchamp (2012); for s5, see Quinto et al. (2010); for s11, see Dodd et al. (2008); Erdener (2015); Erdener and Burnham (2013); for s15, see Skipper et al. (2007); van Wassenhove et al. (2007). For s7 and s13, speech was recorded to Betamax analog videotape (recordings were made in 1997). The talker had an earpiece in the right ear with a click at 120 BPM to equate the syllabic tempo across different recordings. The two component recordings used to create each McGurk stimulus (video and audio) were synchronized by editing the audio track so that the replacement auditory speech commenced at the same time as the first audible sound in the video track whose audio track was being replaced.

For online presentation, all stimuli were encoded using the MPEG-4 AAC encoder with an auditory sampling rate of 48,000 Hz. Videos were presented at a fixed size of 1,300 × 650 pixels.

## Results

### Responses to McGurk stimuli

Across the 15 different McGurk stimuli (each presented 10 times), the 80 participants reported an average of 23% fusion responses (± 1% standard error of the mean across participant). There was a high degree of variability in the number of fusion responses across the 15 different McGurk stimuli: the weakest stimulus evoked the McGurk effect on 3% (± 1%) of trials while fusion responses were evoked on 78% (± 4%) of trials by the strongest stimulus. There was also a high degree of variability in the percentage of McGurk responses across different participants: across stimuli, the least-susceptible participant perceived the illusion on 0% of trials (± 0% standard error of the mean across stimuli), while the most-susceptible participant perceived the illusion on 91% of trials (± 3%). The combination of high stimulus and high participant variability meant that for most of the tested stimuli, the frequency of fusion responses ranged from the lowest possible value of 0% for some participants to the highest possible value of 100% for other participants.

### Model fitting examples

**Figure 2:**
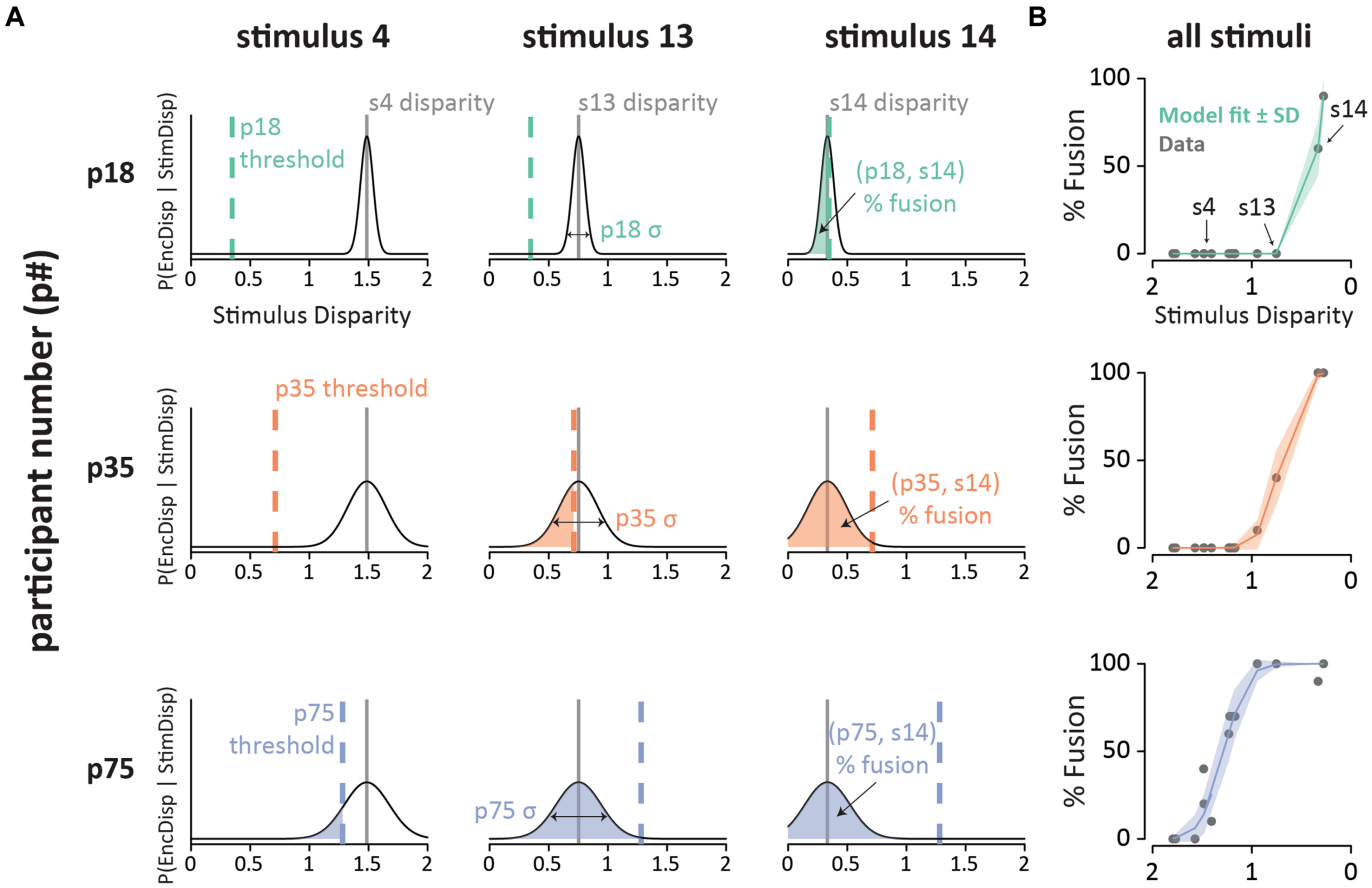
**(A)** Fits of the noisy encoding of disparity (NED) model across participants and stimuli. Each row shows a single participant (p18, p35, p75; one color per participant). Each column shows a different stimulus (s4, s13, s14). The x-axis shows the estimated stimulus disparity. The y-axis shows probability. The thin black line is the Gaussian probability distribution of the disparity estimates. The stimulus disparity is fixed for each stimulus (gray vertical lines). On each presentation of a stimulus, the stimulus disparity is estimated by the observer with sensory noise, σ (horizontal arrows; fixed for each participant). The estimated disparity is compared with the participant’s integration threshold (vertical colored dashed line, fixed for each participant). If the estimated disparity is below the threshold, the participant experiences the McGurk fusion percept. The shaded area is the predicted percent fusion for that participant and stimulus. **(B)** Summary of the model fit for all stimuli for the three participants. The x-axis shows the estimated stimulus disparity, with values reversed to produce an increasing psychometric function. The y-axis shows the % fusion reports for each stimulus. Each gray point shows raw data, colored line shows model prediction (shaded color region shows model SD).

Figure 2A illustrates the model fitting process for three stimuli which evoked different average rates of fusion perception. For each stimulus, the model estimates an audiovisual disparity, which is assumed to be constant (i.e., a physical property of the stimulus). On each presentation of a stimulus, the participant must encode the audiovisual disparity of the stimulus. Due to sensory noise, the encoding is not precise, resulting in a Gaussian distribution of disparity estimates across presentations. The mean of this distribution is the true disparity of the stimulus, and the degree of sensory noise (width of the Gaussian distribution) is estimated by the model separately for each participant. For each stimulus presentation, the participant compares the estimated stimulus disparity with a fixed, internal threshold. If the estimated stimulus disparity exceeds the internal threshold, the participant assumes that the auditory and visual component of the speech comes from different talkers, and perception defaults to the auditory component of the stimulus. If the estimated disparity does not exceed the threshold, the participant integrates the auditory and visual components of the stimulus and perceives the illusion.

For participants with three very different rates of average fusion perception: low (p18; 10%), moderate (p35; 17%), and high (p75; 53%), the model accurately predicted perception across the 15 different McGurk stimuli (Figure 2B). Instead of fitting the NED model, one could simply use the mean fusion rate of each participant as a predictor. However, this approach produces very large errors. For instance, for p18, stimulus 14 evoked 60% fusion percepts, which is identical to the NED model prediction (0% error). In contrast, predicting the mean fusion rate for p18 of 10% produces a 50% error. Similarly, the mean fusion rate for p75 across stimuli is 53%, resulting in a large error if this is used as the prediction for stimulus 1 (0% fusion percepts; 53% error). In contrast, the NED model predicts 0.3% fusion percepts (0.3% error). High error also results if participant variability is ignored, and the mean fusion rate for each stimulus is used for prediction.

### Model fitting

**Figure 3:**
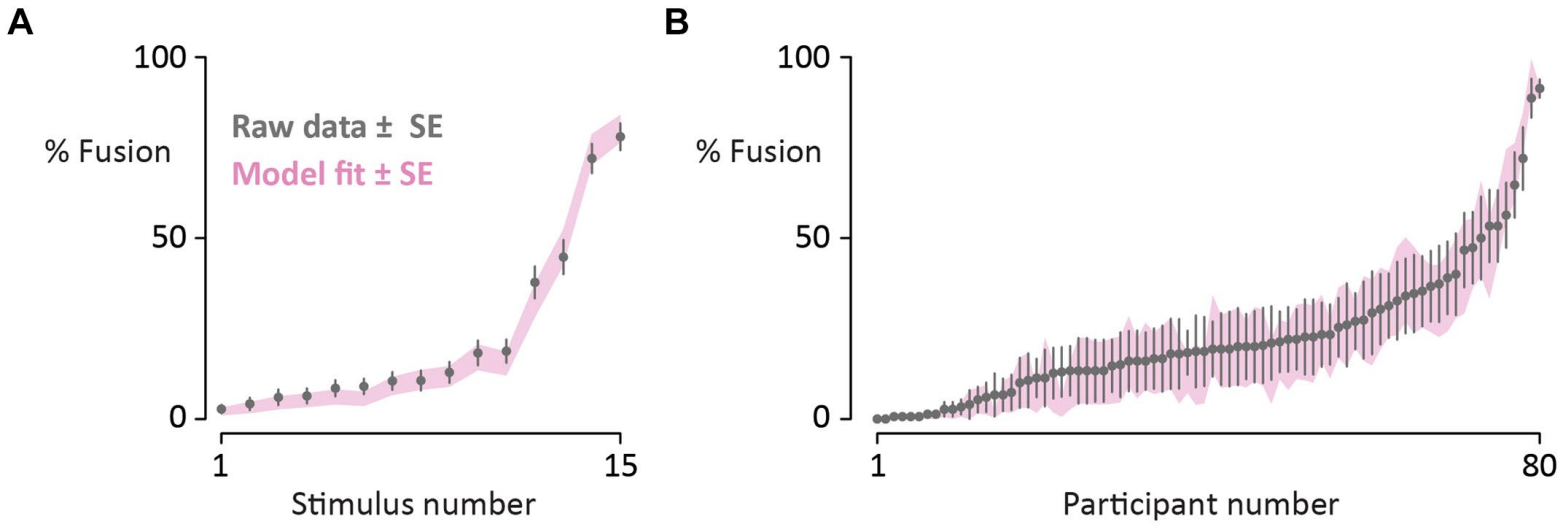
**(A)** Participants reported their percepts of 15 different McGurk stimuli (10 repetitions each, randomly interleaved with congruent speech). Gray circles denote the mean percentage of fusion responses across participants for each McGurk stimulus (raw data; bars show standard error of the mean across participants). Stimuli are sorted from fewest to most fusion responses. The pink shaded region shows the fit of the noisy encoding of disparity model (mean ± one standard error). **(B)** For each of 80 participants, the mean percentage of fusion responses across the 15 different McGurk stimuli was calculated (one gray circle per participant; raw data; bars show standard error of the mean across stimuli; participants sorted from fewest to most fusion responses). The shaded region shows the fit of the noisy encoding of disparity model (mean ± one standard error).

Figure 3 shows the model results across stimuli and participants. The model accurately reproduced stimulus-level variation, predicting stimulus-level mean fusion responses with a mean absolute error of 2.1% ± 0.4% SEM. The model reproduced subject-level variation with an average error of 2.4% ± 0.3%. For single stimulus-participant pairs, the response could be predicted with an average error of 4.7% ± 0.5%.

Validating the model assumption of a constant audiovisual disparity for each stimulus, the stimulus ranks were highly correlated across subjects (average subject-level rank correlation with global rank, *r* = 0.68 ± 0.02, *p* < 10^−16^).

We also estimated out-of-sample generalization using leave-one-stimulus-out model fitting. Fusion percentages on untrained stimuli could be predicted with an error of 9.7% ± 0.5%. The error rate was low for untrained stimuli (9.7% vs. 4.7% for trained data) demonstrating that the high accuracy of the model predictions was not due to overfitting of the training data.

### Stimulus differences

The 15 McGurk stimuli differed along several dimensions, including syllabic composition (the voiced syllables AbaVga vs. the unvoiced syllables ApaVka), the gender of the talker, and the native language of the talkers (Japanese vs. non-Japanese). To test the importance of these factors, we used stepwise linear regression to find the predictors of stimulus disparity as a function of syllable, talker gender, and talker native language. The best-fitting model explained 70% of the variance in the stimulus disparity parameter [*R*^2^ = 0.70, *F*(1, 13) = 30.2, *p* = 10^−4^] and included only syllable content (*b* = −0.92). The mean fusion rate was 65% for voiceless ApaVka stimuli compared with 12% for voiced AbaVga stimuli.

A possible concern is confounding of talker differences and syllable content. This concern was mitigated by the fact that the stimulus set contained examples of both pairings recorded from the same talkers (Magnotti et al., 2024). For talker Audrey Nath, the ApaVka pairing evoked 72% fusion responses while AbaVga evoked 6% fusion responses. For talker Arnt Maasø, ApaVka evoked 45% fusion while AbaVga evoked 11% fusion responses.

Neither talker gender [*F*(1, 11) = 1.2, *p* = 0.3] nor talker language [*F*(1, 11) = 10^−4^, *p* = 0.99] were significant predictors of stimulus-level differences.

### Participant differences

The NED model estimated sensory noise and disparity threshold parameters for each participant. Statistical modeling revealed two significant associations between these parameters and participant demographic variables. There was a significant positive correlation between the sensory noise parameter and age of the participant [*b* = 0.003, *R*^2^ = 0.07, *F*(1, 78) = 5.7, *p* = 0.02], indicating greater sensory noise with increasing age. In contrast, the disparity threshold parameter was not predicted by participant age [*b* = 0.002, *R*^2^ = 0.003, *F*(1, 78) = 0.20, *p* = 0.66].

Conversely, there was a significant negative correlation between self-reported English proficiency rating and the disparity threshold parameter [*b* = −0.07, *R*^2^ = 0.06, *F*(1, 78) = 5.1, *p* = 0.03], while English proficiency was not predictive of the sensory noise parameter [*b* = −0.011, *R*^2^ = 0.02, *F*(1, 78) = 1.9, *p* = 0.17].

## Discussion

The noisy encoding of disparity (NED) model was fit to the perceptual reports of 80 native Japanese speakers presented with 15 different McGurk stimuli. Despite the high variability of the McGurk effect (ranging from 0 to 100% fusion reports across participants for most stimuli), the NED model predicted perception of the illusion with only a few percent error for individual stimuli, participants, and stimulus-participant pairs.

The NED model makes two fundamental assumptions. First, it assumes that individual differences in audiovisual speech perception can be captured by two simple parameters, that of sensory noise and sensitivity to audiovisual disparity. Second, the model assumes that different McGurk stimuli can be characterized by the amount of audiovisual disparity they contain. The ability of the NED model to accurately predict perception in native Japanese speakers, native English speakers, native German speakers, and native German adults with cochlear implants (Magnotti and Beauchamp, 2015; Stropahl et al., 2017) demonstrate that these assumptions are satisfied in three very different participant populations.

To more concretely test the assumption that different McGurk stimuli can be characterized by an intrinsic audiovisual disparity independent of participant native language, we compared the model results for the present study of native Japanese speakers and the native English speakers tested in the original description of the NED model. The stimulus rankings between the two studies were strongly correlated, *r* = 0.87, *p* < 10^−16^, demonstrating the reasonableness of defining an intrinsic disparity for each stimulus.

### Participant variability and intercultural comparisons

In the present study, we found high variability in the McGurk effect across native Japanese speakers, consistent with the high variability observed in studies of native English speakers (Schwartz, 2010; Jiang and Bernstein, 2011; Stevenson et al., 2012; Basu Mallick et al., 2015). The high variability inherent in the McGurk effect *within* single cultures complicates studies of potential differences in the effect *across* cultures. Using simulations, Magnotti and Beauchamp (2018) estimated the number of participants required to detect group differences in the McGurk effect with 80% power, a common statistical benchmark (Cohen, 1992). Even assuming a moderately-sized mean difference of 10% in fusion rate between groups, more than 300 participants would be required to reliably detect this difference.

The large sample size required for well-powered detection of group differences in fusion rates necessitates careful evaluation of published studies: a “statistically significant” finding in an underpowered study may greatly inflate the measured effect-size (Gelman and Weakliem, 2009). Intercultural comparisons of the McGurk effect with larger sample sizes have largely failed to detect any difference in fusion rates. A study with a sample size of 307 did not find a significant difference in fusion rates between native English speakers tested in the USA and native Mandarin speakers tested in China (Magnotti et al., 2015). A study with a sample size of 99 did not find a significant difference in fusion rates between native Finnish speakers and native Japanese speakers (Tiippana et al., 2023). In contrast, a study reporting low rates of McGurk fusion in native Japanese speakers tested just 10 participants (Sekiyama and Tohkura, 1991).

### Stimulus variability

Just as variability across participants complicates intercultural comparisons, so does variability across stimuli. Across the 15 different McGurk stimuli tested in the present study, there was high variability in the rate of fusion percepts, ranging from 3 to 78%. This large variability is problematic when making inferences from only a few stimuli.

For instance, there are mixed reports in the literature about the existence of cross-language influences in the McGurk effect. Some studies report *more* fusion responses when native speakers of one language are presented with McGurk stimuli recorded by a native talker of another language (Ujiie and Takahashi, 2022); other studies report *fewer* fusion responses or mixed results (Chen and Hazan, 2007). In the present study, there was no significant difference in fusion responses between the two stimuli recorded by native Japanese speakers and the other stimuli (recorded by native speakers of other languages).

Given the high variability across McGurk stimuli, attempts to identify differences between stimulus categories with only one or two examples from each category are unlikely to yield reliable results. Instead, it is important to test as many stimuli from each category as possible. The largest study of this type tested Japanese and Finnish participants using McGurk stimuli recorded by four native Japanese talkers and four native Finnish talkers and found no significant difference between Japanese and Finnish talkers (Tiippana et al., 2023).

There are many differences between the 15 McGurk stimuli that we tested, since they have been made at different times by different groups. However, high perceptual variability was also observed across 20 different McGurk stimuli created by the same talker with identical recording and editing methods (Magnotti et al., 2020), suggesting that auditory and visual speech features are the key drivers of variability rather than technical issues (such as analog vs. digital recording). In the present study, the only reliable predictor of the efficacy of different McGurk stimuli was their syllabic composition, with much higher fusion rates for ApaVka than AbaVga.

### Online testing

Data for this study were collected online, which have both advantages and disadvantages. An advantage of online testing is that it is easier to sample participants with a wider range of ages. The NED model was developed with data collected in-person from university students, resulting in a limited participant age range of 17 to 26 years old. In contrast, online participants had a broader range of ages between 18 and 63 years. This larger age range revealed a correlation between age and the sensory noise parameter of the NED model that was not apparent within the limited age range of university students.

A disadvantage of online testing is that compared with in-person testing, it provides less control over stimulus details, such as the size of the visual image or the loudness of the speech stimuli. However, the variability observed in the present study cannot be attributed solely to online testing, as in-person testing revealed similarly high levels of variability (Basu Mallick et al., 2015; Magnotti et al., 2015; Feng et al., 2019). Variability in the McGurk effect also cannot be solely attributed to failure to observe the display, as shown by high variability even when participant eye movements are monitored (Gurler et al., 2015; Rennig et al., 2020; Stacey et al., 2020).

We cannot rule out the possibility that some participants with low fusion rates ignored the task instructions to watch the stimulus videos. However, the cadence of the task was designed to encourage attention to the display; participants were required to click on a “play” button; attend to a ~ 2-s stimulus video; click on a response box; type in what the participant said; and repeat. Therefore, a hypothetical troublemaker would have to look at the display to click the play button and avert their gaze from the brief video while still attending to the auditory portion of the stimulus and then return their gaze to the display to click on the response text box and enter a response corresponding to the auditory portion of the stimulus (if the participant completely ignored the stimulus, the accuracy of their responses to congruent stimuli would be low and they would be excluded from the analysis). Furthermore, they would have to adopt this strategy on some trials but not others, as all but two participants reported the McGurk effect on at least one trial.

Collecting data online could affect the NED model fit in several ways. Since increased auditory noise increases McGurk fusion percepts (Fixmer and Hawkins, 1998; Stacey et al., 2020), online participants who ignored the task instructions to complete the task in a quiet environment and were in a noisy environment would be expected to have higher fusion rates. If online participants were less attentive to the stimuli than in-person participants, this could lead to more variability in responding, manifesting as increased estimates of participant sensory noise or increased error in the NED model fit. Arguing against these scenarios, the disparity threshold and sensory noise parameters were comparable for online participants in the present study and in-person participants in the original description of the model, with less cross-validated fit error (10% for online vs. 19% for in-person).

### Limitations

A limitation of the NED model is that it is primarily descriptive rather than mechanistic. For instance, it takes as a given that participants who integrate auditory “ba” with visual “ga” report the fusion percept of “da” rather than providing an explanation for the fusion percept. Mechanistic models often ignore individual differences but incorporate sequential steps of unisensory estimation and multisensory integration using principles of Bayesian inference (Andersen and Winther, 2020; Lindborg and Andersen, 2021) and causal inference (Magnotti et al., 2013, 2018; Magnotti and Beauchamp, 2017). Fitting mechanistic models require substantially more data, including perceptual measurements of unisensory auditory and visual speech, usually with sensory noise added to each modality at varying levels or different degrees of temporal asynchrony between modalities. In contrast, the simpler NED model only requires measuring the perception of McGurk stimuli.

It remains to be clarified how the parameters in the NED model relate to real-world variables. The model assumes that audiovisual disparity is an intrinsic property of different McGurk stimuli. Perceptual studies using advanced synthetic faces should also allow more insight into understanding and manipulating the factors contributing to stimulus disparity (Thézé et al., 2020; Varano et al., 2021; Shan et al., 2022; Yu et al., 2024), as should measurements of the mouth and face movements made by real talkers (Jiang et al., 2007). The NED model fits a sensory noise parameter for each participant, with the finding that sensory noise increases with age. The variability of the BOLD fMRI response to audiovisual speech also increases with age (Baum and Beauchamp, 2014). This suggests that measuring neural variability in speech processing regions could allow an independent assessment of sensory noise, linking a NED model parameter to brain activity. A recent fMRI study found that observers’ response entropy was greater for McGurk compared with congruent audiovisual stimuli, corresponding to increased BOLD activity in brain regions important for cognitive control (Dong et al., 2024). Parietal and frontal regions are important for causal inference on audiovisual stimuli (Gau and Noppeney, 2016; Mihalik and Noppeney, 2020). Brain activity in these regions could be measured to provide an independent estimate of a participant’s disparity threshold for integrating auditory and visual speech.

## Data availability statement

The original contributions presented in the study are included in the article/Supplementary material, and further inquiries can be directed to the corresponding author.

## Ethics statement

The studies involving humans were approved by the University of Pennsylvania Institutional Review Board. The studies were conducted in accordance with the local legislation and institutional requirements. The ethics committee/institutional review board waived the requirement of written informed consent for participation from the participants or the participants’ legal guardians/next of kin because participants were recruited and tested online. Written informed consent was obtained from the individual(s) for the publication of any potentially identifiable images or data included in this article.

## Author contributions

JM: Writing – original draft, Writing – review & editing. AL: Writing – original draft, Writing – review & editing. MB: Writing – original draft, Writing – review & editing.
