# Supplementary material for: The noisy encoding of disparity model predicts perception of the McGurk effect in native Japanese speakers
Source: Front Neurosci. 2024 Jun 26;18:1421713. doi: 10.3389/fnins.2024.1421713 (PMC11233445; doi:10.3389/fnins.2024.1421713)
Supplement: Supplementary file 1 [file Data_Sheet_1.ZIP › AnalysisCode.html]

The Noisy Encoding of Disparity Model Predicts Perception of the McGurk Effect in Native Japanese Speakers


Code 

- Show All Code
- Hide All Code

# The Noisy Encoding of Disparity Model Predicts Perception of the McGurk Effect in Native Japanese Speakers

#### John F. Magnotti, Anastasia Lado, Michael S. Beauchamp

- 1 Code setup
  - 1.1 helper functions
- 2 Load data
- 3
  Demographic variables
- 4 Assess congruent
  performance
- 5 Analyze McGurk data
  - 5.1 subset McGurk data based on
    Congruent performance
  - 5.2 Get variation in McGurk by
    participants and stimuli
- 6 Fit NED Model
  - 6.1 Functions needed
  - 6.2 Arrange the data
  - 6.3 Fit NED model
  - 6.4 Inspect model fit and
    parameters
    - 6.4.1 Compare stimulus ranks
  - 6.5 NED Model parameters
  - 6.6 Show fits to individual subject
    data
  - 6.7 Inspect subjects with different
    parameters
  - 6.8 Plot fitted data by subject /
    stimulus
  - 6.9 Leave one out analysis
    - 6.9.1 Compare generalization
      performance to training performance
  - 6.10 Control analysis: Participant
    demographics
    - 6.10.1 Regression for threshold
      parameter
    - 6.10.2 Sensory noise
      regression
    - 6.10.3 Model parameter
      dissociation
  - 6.11 Explaining stimulus
    variation
  - 6.12 Control analysis: Correlate
    stimulus ranks with US data (from Magnotti & Beauchamp,
    2015)

# 1 Code setup

```
stopifnot(all(
  sapply(c('lme4', 'car', 'readxl', 'magrittr', 'stringr', 'knitr', 'future', 'future.apply', 'memoise'), 
         require, quietly = TRUE, character.only=TRUE)
))

knitr::opts_chunk$set(eval = TRUE, fig.align = 'center', class.source='fold-hide')
```

## 1.1 helper functions

```
m_se <- function (x) {
  if (length(x) == 1) 
    return(c(mean = x, se = 0))
  
  c(mean = mean(x, na.rm=TRUE), se = se(x))
}

not_NA <- function(x) !is.na(x)

se <- function (x, na.rm = TRUE)  {
  n <- sum(not_NA(x))
  re <- if (n < 2) {
    0
  }
  else {
    stats::sd(x, na.rm = na.rm)/sqrt(n)
  }
  if (is.na(re)) {
    re <- 0
  }
  return(re)
}

# kable doesn't like matrix output from aggregate
fix_agg_columns <- function(m) {
  to_fix <- which(sapply(m, is.matrix))
  for(ii in seq_along(to_fix)) {
    
    mat = m[[to_fix[ii]]]
    for(v in colnames(mat)) {
      m[[paste0(names(to_fix)[ii], '_', v)]] = mat[,v]
    }
  }
  m[to_fix] = NULL
  return(m)
}


# data manipulation
order_by <- function(.data, var, env=parent.frame()) {
  vv = eval(substitute(var), env=.data)
  
  if(is.matrix(vv)) {
    vv = vv[,1]
  }
  .data[order(vv), ]
}

lighten <- function(col, amt=0.5) {
  if(length(col) > 1) return(sapply(col, lighten, amt=amt))
  
  # cc = c(col2rgb(adjustcolor(col, amt), alpha = TRUE))
  
  cc <- c(col2rgb(col))
  
  new_color <- 255 * (
    cc/255 * (amt) + c(1,1,1)*(1-amt)
  )
  
  rgb(new_color[1], new_color[2], new_color[3], maxColorValue = 255)
}

# graphing functions
plot_clean <- function (xlim, ylim=xlim, x = 1, y = 1, type = "n", xlab = "", ylab = "", 
                        ...) 
{
  plot(x, y, type = type, axes = F, ylab = ylab, xlab = xlab, 
       xlim = range(xlim), ylim = range(ylim), ...)
}


ebar_polygon <- function (x, y, sem, alpha = 100/255,
                          col = "black", fill = col, stroke = col,
                          border = NA, add_line = TRUE, lwd = 1, ...) 
{
  is_finite = is.finite(y) & is.finite(sem)
  if (all(!is.finite(sem))) {
    is_finite <- is.finite(y)
    sem <- 0 * y
  }
  x = x[is_finite]
  y = y[is_finite]
  sem = sem[is_finite]
  sem = abs(sem)
  
  polygon(c(x, rev(x)), c(y + sem, rev(y - sem)), border = border, 
          col = adjustcolor(fill, alpha))
  
  if (add_line) 
    lines(x, y, col = stroke, lwd = lwd, ...)
}


ebars.y <- function (x, y, sem, length = 0.05, up = T, down = T, code = 2, ...) {
  if (up) {
    arrows(x0 = x, y0 = as.numeric(y), y1 = as.numeric(y + 
                                                         sem), angle = 90, code = code, length = length, ...)
  }
  if (down) {
    ### make sure the values don't go below 0 for visualization
    y1 = pmax(0, as.numeric(y - sem))
    arrows(x0 = x, y0 = as.numeric(y), y1 = y1, angle = 90, code = code, length = length, ...)
  }
}

ebars <- function (x, y = NULL, sem = NULL, length = 0.05, type = "n", 
                   col = "black", pt.col = col, code = 0, lwd=1, pt.lwd=1, ...) {
  if (is.null(y)) {
    if (is.matrix(x)) {
      y <- x[, 1]
      sem <- x[, 2]
    }
    else {
      y <- x
    }
    x <- seq_along(y)
  }
  if (is.matrix(y)) {
    sem <- y[, 2]
    y <- y[, 1]
  }
  if (is.null(sem)) {
    sem <- y
    y <- x
    x <- seq_along(y)
  }
  ind = (sem>0) & is.finite(sem)
  ebars.y(x[ind], y[ind], sem[ind], length, code = code, col = col, lwd=lwd, ...)
  
  points(x, y, type = type, col = pt.col, lwd=pt.lwd, ...)
}

ruta_axis <- function (side, at, tcl = -0.3, labels = at, las = 1, cex.axis = 1.3, 
                       cex.lab = 1.3, mgpy = c(3, 0.6, 0),
                       mgpx = c(3, 0.75, 0), ...)  {
  if (length(side) > 1) {
    return(invisible(sapply(side, ruta_axis, at = at, tcl = tcl, 
                            labels = labels, cex.axis = cex.axis, las = las, 
                            cex.lab = cex.lab, ...)))
  }
  mgp <- mgpy
  if (side%%2) 
    mgp <- mgpx
  invisible(as.matrix(axis(side, at = at, labels = labels, 
                           tcl = tcl, mgp = mgp, cex.axis = cex.axis, las = las, 
                           cex.lab = cex.lab, ...)))
}

plot_fusion <- function(.data, x=1:nrow(.data), xlim, col='gray45', ..., type='p', pch=16,
                        pt.cex = 1, pt.lwd=1, xlabels, reorder=FALSE, add=FALSE) {
  
  if(isTRUE(reorder)) {
    .data = .data[order(.data$pct_fusion[,1]),]
  }
  
  .data$pct_fusion %<>% as.matrix
  
  if(missing(xlim)) {
    xlim = range(x)
  } 
  
  if(!add) {
    plot_clean(xlim, 0:100, ...)
  }
  points(x, .data$pct_fusion[,1], pch=pch, type=type, cex=pt.cex, lwd=pt.lwd, col=col)
  
  if(missing(xlabels)) {
    xlabels = xlim
  }
  
  ruta_axis(1, xlim, labels=xlabels)
  
  ruta_axis(2, 0:2*50)
  
  if(ncol(.data$pct_fusion)==2) {
    ebars(x, .data$pct_fusion[,1], .data$pct_fusion[,2], col=col)
  }
}


fix_pdf_name <- function (fname){
  if (!grepl("\\.pdf$", fname)) {
    fname = paste0(fname, ".pdf")
  }
  return(fname)
}

as_pdf <- function (fname, w, h, expr, to_pdf = FALSE,
                    bg = "white", env=parent.frame())  {
  sub_expr = substitute(expr)
  
  if (isTRUE(to_pdf)) {
    on.exit(dev.off())
    fname <- fix_pdf_name(fname)
    pdf(fname, width = w, height = h, useDingbats = FALSE, 
        bg = bg)
    
    eval(sub_expr, envir = env)
  }
  
  res = eval(expr)
  
  return(invisible(res))
}
```

# 2 Load data

```
all_data <- readxl::read_excel(
  'datafile.xlsx', 
  sheet=1,
  col_types = c(rep('text',3), 'numeric', 'text', rep('numeric', 5))
)

all_data$pct_auditory = 100 * (all_data$resp_a / all_data$resp_all)
all_data$pct_fusion = 100 * (all_data$resp_f / all_data$resp_all)
all_data$pct_nota = 100 - all_data$pct_auditory

all_data %<>% split((.)$stim_type)
```

# 3 Demographic variables

```
# Note: These data have already been subsetted to remove participants that did not pass the acccuracy 
# filter.
demog <- readxl::read_excel('datafile.xlsx', sheet='demographics')

demog$participant_ID %<>% as.character

kable(table('Gender' = demog$gender))
```

| Gender | Freq |
| --- | --- |
| F | 44 |
| M | 36 |

```
kable(table('Educ' = demog$education))
```

| Educ | Freq |
| --- | --- |
| 2 | 13 |
| 5 | 42 |
| 6 | 15 |
| 7 | 4 |
| 8 | 3 |
| 9 | 3 |

```
kable(table('Language'=demog$language))
```

| Language | Freq |
| --- | --- |
| Japanese | 80 |

```
mean(demog$eng_level)
```

```
## [1] 3.025
```

```
any(1==demog$hearing)
```

```
## [1] FALSE
```

```
any(1==demog$vision)
```

```
## [1] FALSE
```

# 4 Assess congruent performance

```
congruent_performance <- aggregate(
  pct_auditory ~ participant_ID + stim_type + language,
  FUN=mean, data=all_data$congruent
)

threshold <- congruent_performance$pct_auditory >= 90

cat(sum(!threshold), 'removed out of', length(threshold), '\n\t', sum(threshold), 'remain\n')
```

```
## 21 removed out of 101 
##   80 remain
```

```
## congruent performance of remaining subjects
by_subj_cong <- aggregate(
  cbind(pct_fusion, pct_auditory) ~ participant_ID + stim_type + language,
  FUN=mean, data=all_data$congruent,
  subset=participant_ID %in% congruent_performance$participant_ID[threshold]
)

aggregate(
  pct_auditory ~ stim_type + language,
  FUN=function(x) {
    c('mean' = mean(x), 'se' = se(x), 'range' = range(x))
  }, data=by_subj_cong
) %>% fix_agg_columns %>% kable
```

| stim\_type | language | pct\_auditory\_mean | pct\_auditory\_se | pct\_auditory\_range1 | pct\_auditory\_range2 |
| --- | --- | --- | --- | --- | --- |
| congruent | jp | 96.11111 | 0.3087513 | 90 | 100 |

```
# how common were fusion responses on congruent data?
aggregate(
  cbind(resp_f, resp_all) ~ stim_type + language, function(x) {
    c(length(x), sum(x))
  }, data=all_data$congruent,
  subset=participant_ID %in% congruent_performance$participant_ID[threshold]
)
```

```
##   stim_type language resp_f.1 resp_f.2 resp_all.1 resp_all.2
## 1 congruent       jp     2400        2       2400       7200
```

# 5 Analyze McGurk data

## 5.1 subset McGurk data based on Congruent performance

```
mcgurk_data <- subset(
  all_data$mcgurk,
  participant_ID %in% by_subj_cong$participant_ID
)
```

## 5.2 Get variation in McGurk by participants and stimuli

```
# get overall fusion for mcgurk vs. cong stimuli
aggregate(pct_fusion ~ 1, m_se, data=mcgurk_data)
```

```
##   pct_fusion.mean pct_fusion.se
## 1       22.687500      1.043903
```

```
by_stimulus <- aggregate(
  pct_fusion ~ stimulus, m_se, data=mcgurk_data 
) %>% order_by(pct_fusion)

kable(by_stimulus %>% fix_agg_columns)
```

|  | stimulus | pct\_fusion\_mean | pct\_fusion\_se |
| --- | --- | --- | --- |
| 3 | dkbeard\_std\_new\_normalized.mp4 | 2.7500 | 1.196316 |
| 8 | mcgurk\_kuhl\_std\_normalized.mp4 | 4.1875 | 1.779907 |
| 4 | kao\_Bg\_normalized.mp4 | 6.0000 | 2.226139 |
| 1 | bgd4\_normalized.mp4 | 6.3750 | 2.129091 |
| 14 | Quinto\_McGurk\_normalized.mp4 | 8.5000 | 2.285757 |
| 5 | Lor\_Bg\_normalized.mp4 | 9.0000 | 2.183076 |
| 6 | Maaso\_VgaAba\_normalized.mp4 | 10.5000 | 2.491441 |
| 2 | dasmcgurk\_std\_new\_normalized.mp4 | 10.6250 | 2.792076 |
| 15 | Ter\_Bg\_normalized.mp4 | 12.8750 | 2.924113 |
| 11 | Mik\_Bg\_normalized.mp4 | 18.2500 | 3.480393 |
| 10 | McGurk2\_new\_normalized.mp4 | 18.7500 | 3.262634 |
| 9 | mcgurk\_medina\_std\_normalized.mp4 | 37.7500 | 4.447211 |
| 7 | Maaso\_VkaApa\_normalized.mp4 | 44.7500 | 4.762475 |
| 12 | pkt\_normalized.mp4 | 72.0000 | 4.108497 |
| 13 | poeppel\_pkt\_normalized.mp4 | 78.0000 | 3.775336 |

```
# get the range of fusion for each stimulus too
aggregate(
  pct_fusion ~ stimulus, function(x) {
    c('min'=min(x), 'max'=max(x))
  }, data=mcgurk_data 
) %>% fix_agg_columns %>% kable
```

| stimulus | pct\_fusion\_min | pct\_fusion\_max |
| --- | --- | --- |
| bgd4\_normalized.mp4 | 0 | 100 |
| dasmcgurk\_std\_new\_normalized.mp4 | 0 | 90 |
| dkbeard\_std\_new\_normalized.mp4 | 0 | 80 |
| kao\_Bg\_normalized.mp4 | 0 | 100 |
| Lor\_Bg\_normalized.mp4 | 0 | 80 |
| Maaso\_VgaAba\_normalized.mp4 | 0 | 100 |
| Maaso\_VkaApa\_normalized.mp4 | 0 | 100 |
| mcgurk\_kuhl\_std\_normalized.mp4 | 0 | 90 |
| mcgurk\_medina\_std\_normalized.mp4 | 0 | 100 |
| McGurk2\_new\_normalized.mp4 | 0 | 100 |
| Mik\_Bg\_normalized.mp4 | 0 | 100 |
| pkt\_normalized.mp4 | 0 | 100 |
| poeppel\_pkt\_normalized.mp4 | 0 | 100 |
| Quinto\_McGurk\_normalized.mp4 | 0 | 100 |
| Ter\_Bg\_normalized.mp4 | 0 | 100 |

```
by_subject <- aggregate(
  pct_fusion ~ participant_ID + language, m_se, data=mcgurk_data 
) %>% order_by(pct_fusion)

by_subject$mean_fusion = by_subject$pct_fusion[,1]

# fusion range across subjects
range(by_subject$mean_fusion)
```

```
## [1]  0.00000 91.33333
```

```
# stimuli to highlight later
stim_to_highlight <- c('bgd4_normalized.mp4',
                       'Maaso_VkaApa_normalized.mp4', 'pkt_normalized.mp4')

# find some subjects to highlight from low, med, high McGurk
subjects_to_highlight= c('597', '618', '454')

subject_ind = sapply(subjects_to_highlight, function(id) {
  which(id == by_subject$participant_ID)
})
```

# 6 Fit NED Model

## 6.1 Functions needed

```
# Specify the range of critical parameters here.
# if subjects have borderline values but not borderline mcgurk (0 or 1)
# this need to be adjusted
DISPARITY_RANGE = c(0, 2)
THRESHOLD_RANGE = c(0, 2)
SENSORY_NOISE_RANGE = c(1e-3, 1)

# ensure low <= x <= hi
clip.x = function(x, low = 1e-3, high = (1 - 1e-3), range=c(low, high)) {
  
  x[x < range[1]] = range[1]
  x[x > range[2]] = range[2]
  
  return(x)
}

# error functions
sum_squared_error = function(y, y.hat) {
  return(sum((y - y.hat)^2, na.rm = TRUE))
}

sum_abs_error <- function(y, y.hat) {
  return(sum(abs(y - y.hat), na.rm = TRUE))
}

mean_abs_error <- function(y, y.hat, n = sum(not_NA(y)) ) {
  return(
    sum_abs_error(y, y.hat) / n
  )
}
root_mean_squared_error = function(y, y.hat, n = sum(not_NA(y)) ) {
  return(sqrt(sum_squared_error(y, y.hat)/n))
}

rmse_total = function(subjs) {
  return(sum(sapply(subjs, function(s) s$rmse)))
}

total_err = function(subjs) {
  return(sum(sapply(subjs, function(s) s$abs_error)))
}


# ---

model_fit = function(fit_function, model, disparity, ...) {
  if (!is.vector(model)) {
    y.hat = predict.var(model, disparity)
    y = model$y
    
    idx = !is.na(y)
    
    return(fit_function(y[idx], y.hat[idx], ...))
  }
  
  return(sapply(model, function(m) model_fit(fit_function, m, disparity, ...)))
}

optim_help.var = function(par, subj, disparity, err_fun) {
  subj$sd = par[1]
  
  if (length(par) > 1) {
    subj$threshold = par[2]
  }
  
  return(model_fit(err_fun, subj, disparity))
}

optim.subj.p1 = function(y, disparities, n.sim = 100, err_fun) {
  
  # create subject object
  subj = list(threshold = 0, sd = 0, rmse = Inf, y = y)
  class(subj) = "var"
  
  # initial conditions for sensory noise and threshold
  k = round(sqrt(n.sim))
  pars = as.matrix(
    expand.grid(
      A=seq(from=SENSORY_NOISE_RANGE[1], to=SENSORY_NOISE_RANGE[2], length=k),
      B=seq(from=THRESHOLD_RANGE[1], to=THRESHOLD_RANGE[2], length=k)
    )
  )
  
  res = apply(pars, 1, optim, optim_help.var,
              subj = subj, disparity = disparities, err_fun=err_fun)
  
  best_res = res[[which.min(sapply(res, function(r) r$value))]]
  
  subj$sd = best_res$par[1]
  subj$threshold = best_res$par[2]
  
  # we can't just take best_res$value here because we minimize sse and we want RMSE
  # minimizing RMSE would require extra work each pass
  subj$rmse = model_fit(root_mean_squared_error, subj, disparities)
  subj$abs_error = model_fit(mean_abs_error, subj, disparities)
  
  return(subj)
}

# prints out a counter each time f is called
with_counter_and_timer = function(f, end = "unknown") {
  counter__ = 1
  function(...) {
    cat("Starting Run", counter__, "of", end, "\n")
    
    counter__ <<- counter__ + 1
    
    begin = proc.time()
    res <- f(...)
    end = proc.time()
    
    cat("\tElapsed Time: ", as.numeric(end - begin)[3], "\n")
    
    return(res)
  }
}

get_min = function(interval, subjs, idx, ...) {
  optimize(optim_disparity.var, interval, subjs = subjs, idx = idx)$minimum
}

optim_disparity.var = function(disparity, subjs, idx, ...) {
  
  # create a new subj list that only contains the data corresponding to idx
  subjs = lapply(subjs, function(subj) {
    subj$y = subj$y[idx]
    return(subj)
  })
  
  return(sum(model_fit(sum_squared_error, subjs, disparity), na.rm = T))
}

optim.mcg = function(pFmat, n.sim = 8 * 4, n.iter=8, method='sae') {
  
  if (method == 'sae') {
    err_fun = sum_abs_error
  } else {
    err_fun = sum_squared_error
  }
  
  return(optim.var_model(pFmat, n.sim, n.iter, err_fun=err_fun, method=method))
}

optim.var_model = function(pFmat,
                           n.sim, n.iter, err_fun, sample.sd=0.1, method='sse') {
  
  
  # we take up the 1-colMeans(...) because LOW disparity ~> HIGH McG
  disp = matrix(nrow=1, exp(1 - colMeans(pFmat, na.rm = TRUE)) - 1)
  
  #build up some randomized intial conditions that generally accord with the sample ranks
  
  if(n.sim > 1) {
    disparities = t(array(rnorm(ncol(pFmat) * n.sim,
                                mean = disp, sd = sample.sd), c(ncol(pFmat), n.sim-1)))
    disparities[1,] = disp
  } else {
    disparities = disp
  }
  
  # wrap the fitting function with a counter and a timer
  # fit_function = with_counter_and_timer(fit.var_model, n.sim)
  fit_function = fit.var_model
  
  res = future_apply(disparities, 1, fit_function,
                     pFmat = pFmat, n.iter=n.iter, err_fun=err_fun,
                     future.seed = TRUE)
  
  if(method == 'sae') {
    res.best = res[[which.min(sapply(res, function(r) r$err.total))]]
  } else {
    res.best = res[[which.min(sapply(res, function(r) r$rmse.total))]]
  }
  
  return(res.best)
}

fit.var_model = function(disparities, pFmat, err_fun, n.iter = 5) {
  res = list()
  
  subjs = future_apply(pFmat, 1, optim.subj.p1, err_fun=err_fun,
                       disparities = disparities, future.seed=TRUE)
  
  res$subjs = subjs
  res$disparities = disparities
  
  res$rmse.total = rmse_total(subjs)
  res$err.total = total_err(subjs)
  
  for (qq in 1:n.iter) {
    disparities = future_sapply(seq(disparities), get_min, interval=DISPARITY_RANGE, subjs=subjs,
                                future.seed=TRUE)
    
    subjs = future_apply(pFmat, 1, optim.subj.p1, disparities = disparities, err_fun=err_fun,
                         future.seed=TRUE)
    
    if (rmse_total(subjs) < res$rmse.total) {
      res$subjs = subjs
      res$disparities = disparities
      res$rmse.total = rmse_total(subjs)
      res$err.total = total_err(subjs)
    }
    # cat('iter ', qq, ' of ', n.iter, ' RMSE: ', (res$rmse.total), '\n')
  }
  return(res)
}

# P_X(McG) = P(X < subj_thresholdoff); X ~ N(movie_disparityicacy, subj_noise)
#
# Note that we clip the threshold \in THRESHOLD_RANGE and sd \in SENSORY_NOISE_RANGE to make sure the fitting procedure
# does lead to arbitrarily extreme values for the parameters.
# This occurs (for instance) when subjects always (never) report a fusion response
#
predict.var = function(object, disparity, ...) {
  object$threshold = clip.x(object$threshold, range=THRESHOLD_RANGE)
  object$sd = clip.x(object$sd, range=SENSORY_NOISE_RANGE)
  
  return(pnorm(object$threshold, disparity, object$sd, lower.tail = TRUE))
}

predict_with_ebar <- function(object, disparity, n = 10, ...) {
  p = predict.var(object, disparity)  
  q = 1-p
  
  cbind('mu'=n*p, 'sd'=sqrt(n*p*q)) / n
}
```

## 6.2 Arrange the data

```
# we need one column per stimulus, one row per subject
all_stimuli <- unique(by_stimulus$stimulus)

# we need to insert NAs for subjects that are missing certain stimuli
by_sbj <- lapply(split(mcgurk_data, mcgurk_data$participant_ID), function(sbj) {
  mat = matrix(sbj$pct_fusion, nrow=1,
               dimnames = list(sbj$participant_ID[1], sbj$stimulus))
  
  return(mat[,by_stimulus$stimulus,drop=FALSE])
})

subj_by_stim <- do.call(rbind, by_sbj) %>% set_rownames(names(by_sbj))

#order according to global mean, scale to 0:1
subj_by_stim <- subj_by_stim[by_subject$participant_ID, ] * 0.01

# ensure we're ordered, as this will be relied on later
stopifnot(
  all(zapsmall(diff(rowMeans(subj_by_stim)), 10) >= 0) && 
    all(zapsmall(diff(colMeans(subj_by_stim)), 10) >= 0)
)

# ensure we match the original values and data order
all((colMeans(subj_by_stim, na.rm=T) - by_stimulus$pct_fusion[,1]) < 1e-14
    & colnames(subj_by_stim) == by_stimulus$stimulus)
```

```
## [1] TRUE
```

## 6.3 Fit NED model

```
# future::plan(future::multisession())
# 
# mcg_model.abs <- optim.mcg(
#   subj_by_stim,
#   n.sim=48,
#   n.iter=14, method = 'sae'
# )
# 
# saveRDS(mcg_model.abs, 'mcg_model.abs_04_17_2024.rds')
mcg_model.abs <- readRDS('mcg_model.abs_04_19_2024.rds')
```

## 6.4 Inspect model fit and parameters

```
# Calculate error for each stimulus
fitted.abs <- t(sapply(mcg_model.abs$subjs, predict.var, mcg_model.abs$disparities)) %>% set_colnames(
  colnames(subj_by_stim)
)

stim.mean.pred <- colMeans(fitted.abs)

stim.err = 100*m_se(abs(stim.mean.pred - colMeans(subj_by_stim)))
cat(sprintf("Mean absolute error (MAE) for stimuli:\n%s\n",
            round(d=3,stim.err)))
```

```
## Mean absolute error (MAE) for stimuli:
## 2.08
##  Mean absolute error (MAE) for stimuli:
## 0.382
```

```
subj.mean.pred = rowMeans(fitted.abs, na.rm=TRUE) #predicted avg mcgurk per sybject
subj.err = 100*m_se(abs(subj.mean.pred - rowMeans(subj_by_stim)))

cat(sprintf("Mean absolute error (MAE) for subjects:\n%s\n",
            round(d=3,subj.err)))
```

```
## Mean absolute error (MAE) for subjects:
## 2.414
##  Mean absolute error (MAE) for subjects:
## 0.285
```

```
by_subject$mae = sapply(mcg_model.abs$subjs, getElement, 'abs_error')
cat("Mean absolute error (MAE) for individual stimuli across participants:\t",
    round(d=2,100*m_se(by_subject$mae)), "\n")
```

```
## Mean absolute error (MAE) for individual stimuli across participants:     4.67 0.51
```

### 6.4.1 Compare stimulus ranks

```
stim_rank_raw <- rank(by_stimulus$pct_fusion[,1])

non0 <- rowSums(subj_by_stim) > 0

subj_ranks <- t(apply(subj_by_stim, 1, rank, ties='average'))
stim_corrs <- apply(subj_ranks[non0,], 1, function(x) {
  cor(x, stim_rank_raw, method='spearman')
})

m_se(stim_corrs)
```

```
##      mean        se 
## 0.6812129 0.0206954
```

```
t.test(stim_corrs)
```

```
## 
##  One Sample t-test
## 
## data:  stim_corrs
## t = 32.916, df = 77, p-value < 2.2e-16
## alternative hypothesis: true mean is not equal to 0
## 95 percent confidence interval:
##  0.6400031 0.7224227
## sample estimates:
## mean of x 
## 0.6812129
```

## 6.5 NED Model parameters

```
## Model Parameters
disparities = mcg_model.abs$disparities %>% set_names(
  colnames(subj_by_stim)
)

by_subject$threshold = clip.x(sapply(mcg_model.abs$subjs, getElement, 'threshold'),
                              range=THRESHOLD_RANGE)

by_subject$noise = clip.x(sapply(mcg_model.abs$subjs, getElement, 'sd'),
                          range=SENSORY_NOISE_RANGE)

aggregate(
  cbind(threshold,noise, mae) ~ 1, function(x) {
    c('m' = mean(x), 'sd' = sd(x)) %>% round(2)
  }, data=by_subject
)
```

```
##   threshold.m threshold.sd noise.m noise.sd mae.m mae.sd
## 1        0.70         0.45    0.10     0.12  0.05   0.05
```

## 6.6 Show fits to individual subject data

```
fitted_with_err <- lapply(mcg_model.abs$subjs,
                          predict_with_ebar,
                          disparities, n=10)

plot_subject_prediction <- function(sbj, disparity, col='orange') {
  plot_clean(DISPARITY_RANGE, c(0,.5))
  
  abline(v=disparity, lwd=2, lend=3, col='gray60')
  
  ruta_axis(1, at=0:4/2)
  
  abline(v=sbj$threshold, col=col, lwd=3, lend=3, lty=2)
  
  X = 0:400/200
  Y = dnorm(X, mean=disparity, sd = sbj$sd)
  
  # scale the Y to make it visually nicer (just for visualization)
  Y = Y / 10
  while(max(Y) > 0.45) {
    Y = Y * .9
  }
  
  lines(X, Y)
  
  ## fill in the area below the threshold
  ind <- which(X<sbj$threshold)
  
  min_y = 0 #par('usr')[3]
  polygon(x=c(X[ind], rev(X[ind])), y=c(rep(min_y, length(ind)), rev(Y[ind])),
          border = NA, col=adjustcolor(col, .5))
}

palette('Set 2')
sbjs <- mcg_model.abs$subjs[as.character(subjects_to_highlight)]

# fusion for the highlighted subjects
by_subject$mean_fusion[subject_ind]
```

```
## [1] 10.00000 16.66667 53.33333
```

```
subj_by_stim[subject_ind,c(1,4,14)]
```

```
##     dkbeard_std_new_normalized.mp4 bgd4_normalized.mp4 pkt_normalized.mp4
## 597                              0                 0.0                0.6
## 618                              0                 0.0                1.0
## 454                              0                 0.4                0.9
```

```
# get the prediction for participant 75 on S1
predict.var(sbjs[[3]], disparity = mcg_model.abs$disparities[1])
```

```
##           B 
## 0.003231885
```

```
# mapping from subject rank to subject ID
print(subject_ind)
```

```
## 597 618 454 
##  18  34  75
```

```
mapply(function(sbj, col) {
  qq <<- 1
  as_pdf('../figure_pieces/compare_3_stimuli_' %>% paste0(subjects_to_highlight[col]), w=5.5, h=4.25/3, {
    par(mar=c(2,1,2,1), mfrow=c(1,3))
    sapply(disparities[stim_to_highlight], function(d) {
      plot_subject_prediction(sbj=sbj, disparity = d, col=col)
      
      if(qq ==1) {
        abline(v=0)
      }
      qq <<- qq + 1
    })
  })
  cbind('actual'=sbj$y[stim_to_highlight],
        'pred'= predict.var(sbj, disparities[stim_to_highlight]))
  
  
}, sbjs, seq_along(sbjs), SIMPLIFY = FALSE)
```

```
## $`597`
##                             actual          pred
## bgd4_normalized.mp4            0.0 1.008968e-100
## Maaso_VkaApa_normalized.mp4    0.0  1.563650e-14
## pkt_normalized.mp4             0.6  6.000000e-01
## 
## $`618`
##                             actual         pred
## bgd4_normalized.mp4            0.0 1.047921e-06
## Maaso_VkaApa_normalized.mp4    0.4 4.000000e-01
## pkt_normalized.mp4             1.0 9.898906e-01
## 
## $`454`
##                             actual      pred
## bgd4_normalized.mp4            0.4 0.1391110
## Maaso_VkaApa_normalized.mp4    1.0 0.9973425
## pkt_normalized.mp4             0.9 0.9999997
```

## 6.7 Inspect subjects with different parameters

```
sbjs <- mcg_model.abs$subjs[as.character(subjects_to_highlight)]
par(mfrow=c(1,3))
# plot single subject
mapply(function(id, ind, col) {
  # id = subjects_to_highlight[1]
  p <- subset(mcgurk_data, participant_ID == id)
  # order by overall stimulus order
  # set rownames
  # then index based on the sort order in the by_stimulus data frame
  rownames(p) = p$stimulus 
  as_pdf(sprintf('../figure_pieces/subject_%s_bystim', ind), w=1.7, h=1.55, {
    par(mar=c(2.5,2.5,1,.5), cex=.7)
    plot_fusion(p[by_stimulus$stimulus,], xlim=0:2, type='p',
                main=paste0('p', ind), xlab='Stimulus Disparity', ylab='Subject % Fusion',
                x=2-disparities, xlabels = 2:0
    )
    
    mse <- 100*predict_with_ebar(sbjs[[as.character(id)]], disparities)
    
    # disparities need to be sorted here tospo the polygon and line look correct
    ord = order(disparities)
    ebar_polygon(2-disparities[ord], mse[ord,1], mse[ord,2], alpha = .3, col=col)
    
    # abline(col=col, v=
    #         2-subset(by_subject, participant_ID == id)$threshold,
    #     lty=2, lwd=2
    # )
    
    # abline(h=mean(p$pct_fusion), lwd=2, col=col)
  })
}, subjects_to_highlight, subject_ind, 1:3)
```

```
## Warning: Setting row names on a tibble is deprecated.
## Setting row names on a tibble is deprecated.
## Setting row names on a tibble is deprecated.
```

```
## $`597`
## NULL
## 
## $`618`
## NULL
## 
## $`454`
## NULL
```

## 6.8 Plot fitted data by subject / stimulus

```
raw = colMeans(sapply(mcg_model.abs$subjs, `[[`, 'y'), na.rm=TRUE)
ind <- order(raw)
par(mfrow=c(1,2))
as_pdf(fname = '../figure_pieces/fitted_stimulus', w=3.25, h=3, {
  par(mar=c(2,3,1,1))
  plot_clean(seq_along(by_stimulus$stimulus), 0:100)
  fit_se <- 100*apply(fitted.abs, 2, m_se) %>% t
  ebar_polygon(1:15,
               fit_se[by_stimulus$stimulus,1],
               fit_se[by_stimulus$stimulus,2],
               col=4, add_line = FALSE)
  plot_fusion(by_stimulus, pt.cex = 1.5/2, pt.lwd=1.5, add=TRUE, col = 'gray45')
})

as_pdf('../figure_pieces/fitted_subj',  w=5, h=3, { 
  par(mar=c(2,2,1,1))
  
  plot_clean(seq_along(by_subject$participant_ID), 0:100)
  fit_se <- 100*apply(fitted.abs, 1, m_se) %>% t
  ebar_polygon(1:nrow(fit_se), fit_se[ind,1], fit_se[ind,2], col=4, add_line = FALSE)
  
  plot_fusion(by_subject, add = TRUE, pt.cex = 0.75,
              col = 'gray45', pch=16)
})
```

## 6.9 Leave one out analysis

```
# future::plan(future::multisession)

loo <- function(holdout) {
  print(paste('fitting data without subject:', holdout))
  
  # first get the stimulus disparities without using the holdout subject
  newdata = subj_by_stim[-holdout,]
  
  mod <- optim.mcg(newdata, n.sim = 10, n.iter = 5, method='sae')
  
  # print("Done, onto stimulus holdouts")
  
  disp <- mod$disparities
  
  subj_data <- subj_by_stim[holdout,]
  
  # now we iterate over stimuli and get predictions for the subject
  yhat = sapply(seq_along(disp), function(holdout_stim) {
    # holdout_stim = 1
    subj.par = optim.subj.p1(y = subj_data[-holdout_stim],
                             disparities = disp[-holdout_stim], err_fun = sum_abs_error)
    
    # now we get the prediction for the heldout stimulus
    predict.var(subj.par, disparity = disp[holdout_stim])
  })
  
  # return the yhat for the held-out stimuli
  yhat
}

# by_subject_results <- future_sapply(1:nrow(subj_by_stim), future.seed = TRUE, loo)
# saveRDS(by_subject_results, 'by_subject_holdout_results_4-17.RDS')

by_subject_results <- readRDS('by_subject_holdout_results_4-19.RDS')
```

### 6.9.1 Compare generalization performance to training performance

```
orig.err <- 100*abs(subj_by_stim - fitted.abs)
cv.err <- 100*abs(subj_by_stim - t(by_subject_results))
m_se(orig.err)
```

```
##      mean        se 
## 4.7550190 0.3664279
```

```
m_se(cv.err)
```

```
##      mean        se 
## 9.6706680 0.5719413
```

## 6.10 Control analysis: Participant demographics

```
with_demog <- merge(as.data.frame(by_subject[,c(1,4:7)]), demog)
with_demog$gender %<>% factor
with_demog$education %<>% factor
```

### 6.10.1 Regression for threshold parameter

```
lm.thresh <- step(trace=FALSE, object = lm(
  threshold ~ age + gender + eng_level + education, 
  data=with_demog), scope=list(lower=lm(threshold ~ 1, data=with_demog)), k=log(nrow(with_demog))
)
summary(lm.thresh)
```

```
## 
## Call:
## lm(formula = threshold ~ eng_level, data = with_demog)
## 
## Residuals:
##     Min      1Q  Median      3Q     Max 
## -0.7695 -0.3543 -0.0136  0.2652  1.3666 
## 
## Coefficients:
##             Estimate Std. Error t value Pr(>|t|)    
## (Intercept)  0.90559    0.10365   8.737  3.5e-13 ***
## eng_level   -0.06804    0.03011  -2.260   0.0266 *  
## ---
## Signif. codes:  0 '***' 0.001 '**' 0.01 '*' 0.05 '.' 0.1 ' ' 1
## 
## Residual standard error: 0.4425 on 78 degrees of freedom
## Multiple R-squared:  0.06145,    Adjusted R-squared:  0.04941 
## F-statistic: 5.107 on 1 and 78 DF,  p-value: 0.02663
```

```
kable(aggregate(
  cbind(mean_fusion, threshold) ~ eng_level, data=with_demog, mean
))
```

| eng\_level | mean\_fusion | threshold |
| --- | --- | --- |
| 0 | 44.14286 | 1.1341820 |
| 1 | 22.22222 | 0.8177489 |
| 2 | 21.11905 | 0.6422063 |
| 3 | 19.66667 | 0.6927064 |
| 4 | 20.42105 | 0.5753891 |
| 5 | 21.13889 | 0.6500462 |
| 6 | 18.41667 | 0.6416634 |

### 6.10.2 Sensory noise regression

```
lm.noise <- step(trace=FALSE, 
                 object = lm(
                   noise ~ age + gender + eng_level + education, 
                   data=with_demog), scope=list(lower=lm(noise ~ 1, data=with_demog)), 
                 k=log(nrow(with_demog))
)

summary(lm.noise)
```

```
## 
## Call:
## lm(formula = noise ~ age, data = with_demog)
## 
## Residuals:
##      Min       1Q   Median       3Q      Max 
## -0.12628 -0.08516 -0.04743  0.07570  0.41566 
## 
## Coefficients:
##             Estimate Std. Error t value Pr(>|t|)  
## (Intercept) 0.012301   0.039408   0.312    0.756  
## age         0.003070   0.001281   2.396    0.019 *
## ---
## Signif. codes:  0 '***' 0.001 '**' 0.01 '*' 0.05 '.' 0.1 ' ' 1
## 
## Residual standard error: 0.1139 on 78 degrees of freedom
## Multiple R-squared:  0.06858,    Adjusted R-squared:  0.05663 
## F-statistic: 5.743 on 1 and 78 DF,  p-value: 0.01895
```

#### 6.10.2.1 Plot between sensory noise and age

```
plot_clean(with_demog$noise, c(18,with_demog$age))
points(with_demog$noise, with_demog$age, pch=16)

ruta_axis(1, c(0,.2, .4))
ruta_axis(2, c(18, 30, 60))
```

### 6.10.3 Model parameter dissociation

```
#show that eng_level doesn't work for noise and age doesn't work for threshold
summary(
  lm(threshold ~ age, data=with_demog)
)
```

```
## 
## Call:
## lm(formula = threshold ~ age, data = with_demog)
## 
## Residuals:
##      Min       1Q   Median       3Q      Max 
## -0.71319 -0.37177  0.05874  0.29344  1.31420 
## 
## Coefficients:
##             Estimate Std. Error t value Pr(>|t|)    
## (Intercept) 0.633299   0.157843   4.012 0.000137 ***
## age         0.002283   0.005131   0.445 0.657650    
## ---
## Signif. codes:  0 '***' 0.001 '**' 0.01 '*' 0.05 '.' 0.1 ' ' 1
## 
## Residual standard error: 0.4562 on 78 degrees of freedom
## Multiple R-squared:  0.002531,   Adjusted R-squared:  -0.01026 
## F-statistic: 0.1979 on 1 and 78 DF,  p-value: 0.6576
```

```
summary(
  lm(noise ~ eng_level, data=with_demog)
)
```

```
## 
## Call:
## lm(formula = noise ~ eng_level, data = with_demog)
## 
## Residuals:
##      Min       1Q   Median       3Q      Max 
## -0.12279 -0.08525 -0.04819  0.07378  0.41504 
## 
## Coefficients:
##              Estimate Std. Error t value Pr(>|t|)    
## (Intercept)  0.134717   0.027314   4.932 4.49e-06 ***
## eng_level   -0.010924   0.007934  -1.377    0.173    
## ---
## Signif. codes:  0 '***' 0.001 '**' 0.01 '*' 0.05 '.' 0.1 ' ' 1
## 
## Residual standard error: 0.1166 on 78 degrees of freedom
## Multiple R-squared:  0.02372,    Adjusted R-squared:  0.01121 
## F-statistic: 1.895 on 1 and 78 DF,  p-value: 0.1725
```

## 6.11 Explaining stimulus variation

```
by_stimulus$disparity = disparities

by_stimulus$syllable = ifelse(
  by_stimulus$stimulus %in% c("pkt_normalized.mp4", "poeppel_pkt_normalized.mp4", "Maaso_VkaApa_normalized.mp4"),
  'pa', 'ba')


by_stimulus$talker = by_stimulus$stimulus
by_stimulus$talker[by_stimulus$stimulus %in% c("pkt_normalized.mp4", "bgd4_normalized.mp4")] = 'audrey'

by_stimulus$talker[by_stimulus$stimulus %in% c("Maaso_VkaApa_normalized.mp4", "Maaso_VgaAba_normalized.mp4")] = 'arnt'

by_stimulus$talker_gender = 'M'
female_talkers = c("poeppel_pkt_normalized.mp4","pkt_normalized.mp4",
                   "Lor_Bg_normalized.mp4", "Quinto_McGurk_normalized.mp4",
                   "bgd4_normalized.mp4", 
                   "kao_Bg_normalized.mp4", "mcgurk_kuhl_std_normalized.mp4")
by_stimulus$talker_gender[by_stimulus$stimulus %in% c(female_talkers)] = 'F'

jp_stim <- c('kao_Bg_normalized.mp4', 'Ter_Bg_normalized.mp4')

by_stimulus$talker_jp <- ifelse(
  by_stimulus$stimulus %in% jp_stim, 'JP', 'other'
)

lm.disparity <- step(trace=FALSE, 
                     object = lm(disparity ~ syllable + talker_gender + talker_jp, data=by_stimulus),
                     scope=list(lower=lm(disparity ~ 1, data=by_stimulus)), 
                     k=log(nrow(by_stimulus))
)
summary(lm.disparity)
```

```
## 
## Call:
## lm(formula = disparity ~ syllable, data = by_stimulus)
## 
## Residuals:
##     Min      1Q  Median      3Q     Max 
## -0.4264 -0.1731 -0.1218  0.1575  0.4243 
## 
## Coefficients:
##             Estimate Std. Error t value Pr(>|t|)    
## (Intercept)  1.37188    0.07469  18.367 1.11e-10 ***
## syllablepa  -0.91774    0.16702  -5.495 0.000103 ***
## ---
## Signif. codes:  0 '***' 0.001 '**' 0.01 '*' 0.05 '.' 0.1 ' ' 1
## 
## Residual standard error: 0.2587 on 13 degrees of freedom
## Multiple R-squared:  0.699,  Adjusted R-squared:  0.6759 
## F-statistic: 30.19 on 1 and 13 DF,  p-value: 0.000103
```

```
aggregate(pct_fusion[,1] ~ syllable, 
          FUN=mean,
          data = by_stimulus)
```

```
##   syllable pct_fusion[, 1]
## 1       ba        12.13021
## 2       pa        64.91667
```

```
aggregate(pct_fusion[,1] ~ syllable + talker, 
          FUN=mean,
          data = by_stimulus, 
          subset=talker %in% c('arnt', 'audrey'))
```

```
##   syllable talker pct_fusion[, 1]
## 1       ba   arnt          10.500
## 2       pa   arnt          44.750
## 3       ba audrey           6.375
## 4       pa audrey          72.000
```

```
# what if you try to fit the other parameters anyway

car::Anova(
  lm(disparity ~ syllable + talker_gender + talker_jp, data=by_stimulus)
)
```

```
## Anova Table (Type II tests)
## 
## Response: disparity
##                Sum Sq Df F value    Pr(>F)    
## syllable      2.01661  1 28.3170 0.0002443 ***
## talker_gender 0.08634  1  1.2124 0.2943614    
## talker_jp     0.00002  1  0.0003 0.9871149    
## Residuals     0.78337 11                      
## ---
## Signif. codes:  0 '***' 0.001 '**' 0.01 '*' 0.05 '.' 0.1 ' ' 1
```

## 6.12 Control analysis: Correlate stimulus ranks with US data (from Magnotti & Beauchamp, 2015)

```
us_mcg <- c('bgd4_normalized.mp4' = 0.1663, 'dasmcgurk_std_new_normalized.mp4' = 0.483, 
            'dkbeard_std_new_normalized.mp4' = 0.2539, 'kao_Bg_normalized.mp4' = 0.2848, 
            'Lor_Bg_normalized.mp4' = 0.1727, 'Maaso_VgaAba_normalized.mp4' = 0.4506, 
            'mcgurk_kuhl_std_normalized.mp4' = 0.1958, 'mcgurk_medina_std_normalized.mp4' = 0.5812, 
            'McGurk2_new_normalized.mp4' = 0.4855, 'Mik_Bg_normalized.mp4' = 0.4258, 
            'pkt_normalized.mp4' = 0.5479, 'poeppel_pkt_normalized.mp4' = 0.8058, 
            'Quinto_McGurk_normalized.mp4' = 0.3227, 'Ter_Bg_normalized.mp4' = 0.4167
)

in_common <- intersect(names(us_mcg), by_stimulus$stimulus)

jp_stim_mean = colMeans(subj_by_stim)[in_common]

cor.test(us_mcg[in_common], jp_stim_mean, method='spearman')
```

```
## 
##  Spearman's rank correlation rho
## 
## data:  us_mcg[in_common] and jp_stim_mean
## S = 58, p-value < 2.2e-16
## alternative hypothesis: true rho is not equal to 0
## sample estimates:
##       rho 
## 0.8725275
```
